# Epidemiology of idiopathic pulmonary fibrosis: a population registry-based study

**DOI:** 10.1186/s13023-026-04210-y

**Published:** 2026-01-19

**Authors:** Gorini Francesca, Santoro Michele, Pierini Anna, Cameli Paolo, Chimera Davide, Lavorini Federico, Pistelli Francesco, Rosi Elisabetta, Tavanti Laura, Tomassetti Sara, Laura Carrozzi, Bargagli Elena, Coi Alessio

**Affiliations:** 1 Unit of Epidemiology of Rare Diseases and Congenital Anomalies, Institute of Clinical Physiology, National Research Council, Pisa, Italy; 2 Foundation Gabriele Monasterio CNR-Regione Toscana, Pisa, 56124 Italy; 3 Department of Medicine, Surgery and Neurosciences, Respiratory Diseases Unit, University of Siena, Siena, Italy; 4 Pulmonary Unit, Cardiothoracic and Vascular Department, Pisa Hospital and University of Pisa, Pisa, Italy; 5 Department of Experimental and Clinical Medicine, Careggi University Hospital and University of Florence, Florence, Italy; 6 Pulmonary Unit, Careggi University Hospital, Florence, Italy; 7 Interventional Pulmonology Unit, Careggi University Hospital, Florence, Italy; 8 Department of Surgical, Medical and Molecular Pathology and Critical Care Medicine, University of Pisa, Pisa, Italy

**Keywords:** Idiopathic pulmonary fibrosis, Prevalence, Incidence, Survival, Population registry-based study

## Abstract

**Background:**

Idiopathic pulmonary fibrosis (IPF) is a chronic and progressive respiratory rare disease characterized by an irreversible loss of lung function, with unknown etiology and poor prognosis.

**Methods:**

A population registry-based study was conducted to provide estimates of prevalence, incidence and survival of IPF. The study included all cases diagnosed with IPF in the years 2000–2022 and residing in Tuscany, Italy. Prevalence as of December 31, 2022, was calculated by sex and age class. Incidence was calculated across the period 2018–2022. Survival at 1, 5 and 10 years from diagnosis with 95% confidence intervals (CI) was estimated by sex and age class using the Kaplan-Meyer method. The independent effect of sex and age at diagnosis on survival was estimated by Cox proportional hazard model.

**Results:**

A total of 1,388 subjects with IPF were diagnosed during the study period. The prevalence as of 31st December 2022 was 21.5 cases (95%CI: 20.0–23.0) per 100,000 inhabitants, with a significantly higher frequency in men and in 70–79 years age group (*p* < 0.0001). The average annual incidence was 4.6 cases per 100,000 inhabitants, with significantly higher incident cases among males (*p* < 0.0001). Survival at 1, 5 and 10 years from diagnosis was 91.3%, 51.4% and 22.2%, respectively. Women exhibited a longer survival than men (34.8% vs. 17.3% at 10 years), while patients under 70 had the highest survival rate at 58.2% (95% CI: 52.5–63.4) at five years. Cox regression model confirmed a higher risk of mortality for men (adjusted Hazard Ratio – adjHR = 1.52, 95%CI: 1.25–1.84, *p* < 0.0001) and with age at diagnosis (adjHR = 1.04, 95%CI: 1.03–1.05, *p* < 0.0001).

**Conclusions:**

The higher prevalence and incidence rates of IPF among men, as indicated by this population registry-based study, align with recent data reported in Europe. Furthermore, IPF is consistently identified as a disease with a poor prognosis, especially in male patients. Therefore, early and accurate diagnosis, coupled with timely management, is essential to improve the care and treatment outcomes for these patients.

## Introduction

Idiopathic pulmonary fibrosis (IPF), accounting for 20% to half of all cases of the broader group of fibrotic interstitial lung diseases [1], is defined as a specific form of chronic, progressive and usually fatal fibrosing interstitial pneumonia, more common in men and elderly patients over 60 years of age [2]. The etiology of IPF is unknown, however it has been postulated that the fibrotic process in IPF is the result of multiple interacting genetic and environmental exposures, usually associated with a history of cigarette smoking, which is considered the strongest risk factor after gene mutations and polymorphisms [1, 3, 4].

IPF is clinically characterized by a progressive and irreversible decline in lung function, primarily resulting to fibrotic distortion of lung parenchyma, which manifests through respiratory symptoms such as shortness of breath, fatigue and chronic dry cough [4, 5]. Despite advancements in therapies, the prognosis of IPF remains poor [5]. However, various phenotypes can be observed in the progression of the disease among different patients [6]. Consequently, alongside the misinterpretation of initial clinical symptoms, the clinical course of IPF can be unpredictable [7, 8].

Based on the definition in force in the European Union, IPF is considered rare (ORPHACODE: 2032), as affecting no more than 1 person in 2,000 [9]. In Europe, 40,000 incident cases of IPF are diagnosed each year, and similar estimates have been recorded in the United States [8, 10].

Over time, IPF epidemiology has been investigated through both single-center [11, 12] and multicenter studies [2, 4, 5, 13–18]. Only a limited number of studies have employed a population-based design covering a defined geographic area or a representative population thereof [19–22]. Among these, none- [19, 21, 22] has simultaneously provided estimates of prevalence, incidence, and survival. Therefore, the aim of this study is to generate accurate and reliable prevalence, incidence and survival estimates (at one, five and ten years) for IPF, exploiting data of a population-based registry that encompasses the entire population residing in the Tuscany (Italy) over a 23-year period (2000–2022).

## Methods

### Study design and study population

We performed a retrospective cohort study including patients diagnosed with IPF in the years 2000–2022 and residing in Tuscany, an Italian region with a population of 3,663,191 inhabitants (source: Italian National Institute of Statistics as of 1 January 2022). The cases were selected from the Registry of Rare Diseases of Tuscany (RRDT), a population registry collecting data from patients resident in Tuscany and diagnosed with a rare disease (in the list of the Italian Decree of the President of the Council of Ministers, 01/2017), which represents one of the main contributors to the Italian National Registry of Rare Diseases [23].

Medical professionals belonging to the Referral Centers of the regional network of rare diseases are the only ones authorised to register cases in the RRDT.

### Study outcomes: prevalence, incidence and survival

Prevalence, expressed as the number of residents diagnosed with IPF per 100,000 inhabitants, was calculated as of 31st December 2022, overall, by sex and by age group (< 70, 70–79, ≥ 80 years). Incidence (expressed as the number of newly diagnosed IPF patients per 100,000 inhabitants) was calculated across the last 5-year period (years 2018–2022). For all subjects diagnosed with IPF in the years 2000–2022, survival estimates were also determined by sex and age class.

### Statistical analysis

Categorical variables (sex, age class) were expressed as counts and percentages, while continuous variables (age) as mean and standard deviation. Differences in data of patients were evaluated with Pearson χ2 test/Fisher’s exact test for nominal and discrete variables and Student’s t test for continuous variables. A Poisson regression model was tested for differences in prevalence by sex and between age groups.

Survival estimates at 1, 5 and 10 years from IPF diagnosis were calculated by sex and age class (< 70, ≥ 70-<80, ≥ 80 years) using the Kaplan–Meier method. The statistical significance of the difference between groups was calculated with the log-rank test. A Cox proportional hazard model was applied to assess the independent impact of sex and age at diagnosis on the risk of mortality. Adjusted hazard ratios (HRs) with 95%CI were provided.

For all statistical tests, a two-sided *p* = 0.05 was considered as statistically significant. The data were analysed with Stata, version 16 [24].

## Results

### Prevalence and incidence

A total of 1,388 cases diagnosed with IPF (1,041 males, 347 females; sex ratio = 3.0) were recorded in the years 2000–2022. IPF patients showed a significant difference by sex across age classes (*p* < 0.0001) (Table 1).

**Table 1:** Distribution by age class of cases with an IPF diagnosis in the years 2000–2022

Age class	Females		Males		Total	
	*N*	%	*N*	%	*N*	%
< 60	49	42.6	66	57.4	115	8.3
60–64	38	27.1	102	72.9	140	10.1
65–69	62	28.6	155	71.4	217	15.6
70–74	69	22.5	237	77.4	306	22.0
75–79	80	22.3	279	77.7	359	25.9
≥ 80	49	19.5	202	80.5	251	18.1
Total	347	25.0	1041	75.0	1388	100

The prevalence as of 31st December 2022 was 21.5 (95%CI: 20.0–23.0]) per 100,000 inhabitants with a significantly higher frequence (*p* < 0.0001) in men (31.9 per 100,000, 95%CI 29.3–34.7) than in women (11.6 per 100,000, 95%CI: 10.1–13.3). Significant differences in prevalence (*p* < 0.0001) were also observed across age classes, with the highest prevalence measured in 70–79 years age group (99.6 per 100,000, 95%CI: 90.2-109.8), followed by the age groups ≥ 80 years (41.3 per 100,000 95%CI: 34.7–48.9) and < 70 years (8.4 per 100.000, 95%CI: 7.4–9.5). In the period 2018–2022, the average annual incidence was 4.6 cases per 100,000 inhabitants; significant differences (*p* < 0.0001) were found between males and females (9.0 and 2.3 per 100,000, respectively).

The mean age at diagnosis was 72.4 ± 9.3 years, with a significant difference (*p* = 0.0005) between males (73.1 ± 8.3 years) and females (70.2 ± 11.8 years).

### Survival

In the period 2000–2022, 592 (465 males, 127 females; *p* = 0.005) out of 1,388 IPF patients died (33.6%). The mean age at death was 74.5 years, with a mean survival from diagnosis of 42.2 ± 37.4 months.

**Fig. 1:**
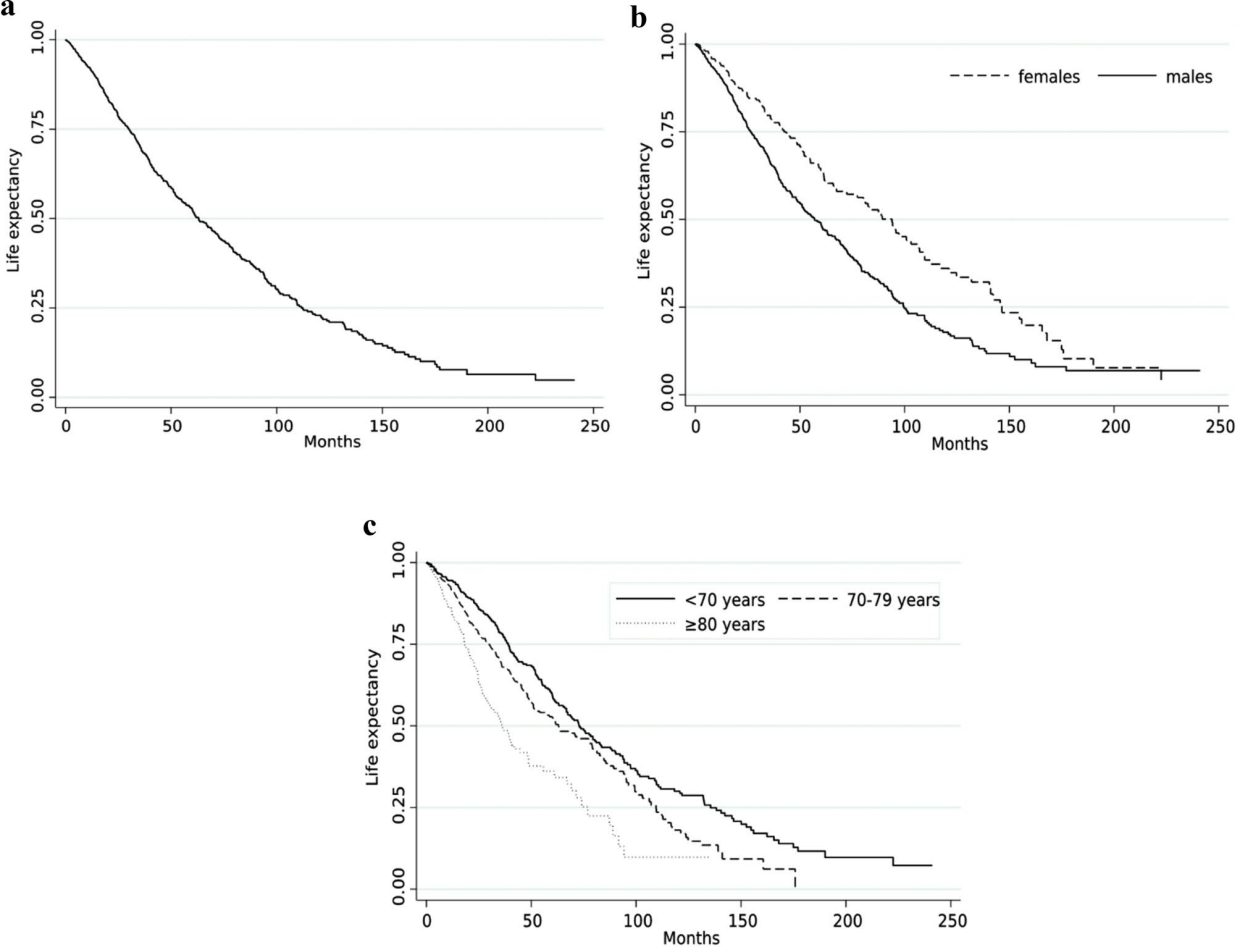
Kaplan–Meier survival estimates for the total of cases (**a**), by sex (**b**), and by age class (**c**)

The overall survival and survival by sex and age classes are depicted in Fig. 1a-c. Survival at one, five and tenyears from diagnosis was 91.3% (95%CI: 89.6–92.7), 51.4% (95%CI: 47.9–54.9) and 22.2% (95%CI: 18.2–26.4), respectively (Fig. 1A).

Women exhibited a significantly longer life expectancy than men (*p* < 0.0001) At five years from diagnosis, survival was 63.9% (95%CI: 56.8–70.3) in women compared to 47.3% (95%CI: 43.4–51.3) in men. The differences widened over time, with 34.8% (95%CI: 26.3–43.5) of women and 17.3% (95%CI: 13.0-22.2) of men still alive at ten years (Fig. 1B).

Significant differences in survival (*p* < 0.0001) were also observed by comparing age classes. At five years, patients under 70 had the highest survival rate at 58.2% (95% CI: 52.5–63.4), followed by those aged 70–79 at 51.4% (95% CI: 46.1–56.5), while individuals aged 80 and above showed the lowest rate at 36.1% (95% CI: 27.3–44.9). Ten years after diagnosis, only 9.8% of patients in the oldest group remained alive (Fig. 1c).

Cox regression analysis confirmed a significantly higher risk of mortality for men, also after adjustment for age at diagnosis (adjHR = 1.52 95%CI: 1.25–1.84, *p* < 0.0001). Mortality also significantly increased with age at diagnosis also after adjustment for sex (adjHR = 1.04: 95%CI: 1.03–1.05, *p* < 0.0001), In particular, subjects aged 80 years or older had a 2.3-fold increased risk of mortality compared to non-elderly patients.

## Discussion

To the best of our knowledge, this is the first populationbased study in Europe to simultaneously report prevalence, incidence, and survival estimates of IPF, encompassing the entire population of Tuscany, a region with 3,663,191 residents, over a 23-year period. Most existing studies on the epidemiology of IPF primarily rely on data retrieved from national registries or administrative databases [2, 4, 13, 14, 16–18, 25–30], while relatively few investigations have employed a trulypopulation-based design [19, 21, 22]. Among these [19, 21, 22], however, none utilized a population-based registry covering the entire resident population of a geographically defined area.Although the incidence of IPF appears to be increasing due to an aging population, a greater degree of disease awareness and improved diagnostic tools [5, 31], there is a substantial heterogeneity in epidemiological estimates of IPF between studies. This variation is likely attributable to differences in classification terms (i.e., inclusion of cases diagnosed before the first consensus statement on IPF) [32] and data collection methods for case ascertainment (e.g., the use of different diagnostic International Classification of Diseases codes and administrative databases such as hospital discharges and mortality registries) [4, 5, 33].

In this study, we observed an overall prevalence of 21.5 cases per 100,000, which is consistent with the estimate previously reported by Agabiti et al. (25.6 per 100,000) [19].

In contrast, the incidence of IPF in Tuscany, equal to 4.6 cases per 100,000 per year, is lower than that national estimate for Italy (7.5 per 100,000 persons per year) shown in [19], likely due to different methods of case ascertainment [19].

The mean age at diagnosis in our cohort (72.4 years) is comparable to that shown in other studies, based on administrative databases [20, 27] or patients registries [13, 30], with men significantly older than female patients, as in the study by Zaman et al. [18].

Our study identified a higher prevalence of IPF among males compared to females, aligning with the existing literature [17, 18, 30, 34]. The basis for this effect is uncertain, but it is hypothesized that male adults are more frequently exposed to cigarette smoke or pollutants in occupational settings (e.g., asbestos, paint, chemicals, metal dust) [3, 4]. Additionally, reproductive hormones might exert different actions in the two sexes [18, 22]. We also observed a significantly higher IPF incidence among men, with a male-to-female incidence rate ratio consistent with findings from a cohort study base on hospital discharge data [22].

Significant differences were also found in the prevalence across age classes, with the majority of patients in both sexes falling within the 70–74 age group. These findings are consistent with those of a previous Italian population-based study [19], which reported peak prevalence among individuals aged 65–74 years of both sexes.

### Survival

This study confirms the extremely high mortality in IPF patients. The mean survival time in our cohort from diagnosis – 3.5 years – is in accordance with the literature [16, 18, 20, 28], while the mean age at death (74.5 years) is slightly higher than that reported by Guenther et al. (71 years), based on data from the European IPF Registry [2]. These differences can be attributable to the different definition for selecting IPF populations and disease severity [22], as well as to under-recognition and misdiagnosis of this condition [7]. The study period may also influence survival, as the approval of antifibrotic medications for IPF treatment (as for Europe, pirfenidone in 2011 and nintedanib in 2015) [35, 36], has been associated with longer patient survival with IPF [13, 30, 37–40].

Male patients were significantly associated with a 52% higher risk for death, after adjustment for age at diagnosis. This is consistent with other recent studies [18, 41], further confirming the worse clinical prognosis of IPF in men compared to women.

Older age at diagnosis also confers a poorer prognosis, as previously reported [13, 20, 30, 39, 42], with a 2.3-fold increased mortality risk in elderly patients compared to those aged < 70 years. A growing body of evidence indicates that accelerated mechanisms of ageing such as oxidative stress, telomere length regulation, changes in a number of antiaging molecules and the extracellular matrix as well as cellular senescence (i.e., the progressive accumulation of senescent fibroblasts and alveolar epithelial cells), contribute to the pathogenesis of IPF [43, 44]. Indeed, circulating biomarkers of cellular senescence, are not only significantly elevated in subjects affected by IPF, but are significantly correlated with measures of pulmonary function, physical function, and risk of death after adjustment for age, sex and body mass index [45]. On the other hand, ablation of cells expressing tumour suppressors, which play a critical role in inducing and maintaining permanent cell cycle arrest during cellular senescence, ameliorates aging-associated lung hypofunction in mice [46].

### Strengths and limitations

The main strength of this study lies in the population-based registry design, which enables the estimation of accurate epidemiological indicators yielding more reliable and generalizable results than hospital-based studies. In addition, this study collected reliable information on more than 1,300 patients diagnosed with IPF across a period of 23 years, which represents a significant achievement for a rare disease, enhancing the statistical power of the findings. Furthermore, compared to the other published population-based studies, our analysis spans a broader and more recent time frame.

Conversely, unlike clinical records, populations registries do not collect data on lifestyle, comorbid conditions, symptoms, lung function, therapies, or diagnostic procedures. Therefore, this study was unable to assess disease severity of patients or adjust survival estimates for potential risk factors comorbidities, and therapeutic regimens.

## Conclusion

This population registry-based study is the first European investigation to provide accurate estimates of prevalence, incidence and survival of one of the most clinically relevant rare respiratory diseases, based on a large cohort over a study period of up to 23 years. IPF is confirmed as a rare disease characterized by a higher prevalence and incidence among men and with poor survival outcomes, especially in male patients. Studies based on population-based registries with long-term data collection are particularly valuable in the field of rare diseases, where accurate epidemiological indicators are often difficult to obtain, especially when cases are derived from hospital-based disease registries that do not cover a defined geographic area.

Overall, beyond its value to public health surveillance, the observed low survival underscore the importance of timely diagnosis and close monitoring of IPF patients, supported by effective interventions and management plans.

## Data Availability

The data that support the findings of this study are available from Regione Toscana, but restrictions apply to the availability of these data, which were used under license for the current study, and so are not publicly available. Data are however available from the authors upon reasonable request and with permission of Tuscany Region.
